# Diagnostic groups of hospital stays and outpatient visits during 10 years before Alzheimer’s disease

**DOI:** 10.1186/s12913-023-09345-3

**Published:** 2023-04-04

**Authors:** Kiira Mäklin, Pasi Lampela, Julian Lin, Hartikainen Sirpa, Anna-Maija Tolppanen

**Affiliations:** Kuopio Research Center of Geriatric Care, School of Pharmacy, University of Eastern Finland, P.O. Box 1627, Kuopio, 70211 Finland

**Keywords:** Alzheimer’s disease, Healthcare use, Hospitalizations, Comorbidities

## Abstract

**Background:**

Alzheimer’s disease (AD) is a major determinant of healthcare costs and increase in the healthcare service use occur already before the AD diagnosis. However, little is known how the different diagnosis categories contribute to this increase in healthcare use. We investigated how the hospitalizations and specialized healthcare outpatient visits from different diagnosis categories, based on the International Classification of Diseases (ICD-10) chapters, contribute to increased specialized healthcare service use during ten-year period preceding AD diagnosis.

**Methods:**

A register-based nationwide cohort of 42,934 community-dwelling persons who received clinically verified AD diagnosis in between 2008 and 2011 in Finland and 1:1 age, sex and hospital district- matched comparison cohort were included. Hospitalizations and specialized healthcare visits were categorized by the main diagnosis, according to the ICD-10 chapters. AD and dementia were separated to their own category. The number of persons with visits and stays was calculated for every 6 months, irrespective of the frequency of visits/stays individual had during that time window. Furthermore, the relative distribution of the diagnosis categories was computed.

**Results:**

AD cohort was more likely to have visits and stays during the 10-year period (OR 1.19, 95% CI 1.17–1.21). The number of persons with visits and stays peaked in AD cohort from 1.5 years before the diagnosis when the differences in relative distribution of different diagnosis categories also became evident. The largest differences were observed for visits/stays with cognitive disorders, symptoms of unspecified diseases and psychiatric disorders diagnoses, and those with missing diagnosis codes in the last time window before AD diagnosis.

**Conclusions and implications:**

Increased healthcare service use before AD diagnosis does not seem to arise from differences in specific diagnosis categories of ICD-10 such as diseases of the circulatory system, but from the higher frequency of visits and stays among persons with AD across diagnosis categories. Based on the relative distribution of diagnosis categories, the steep increase in healthcare service use just before and during the diagnostic process is likely due to prodromal symptoms and visits related to cognition.

**Supplementary Information:**

The online version contains supplementary material available at 10.1186/s12913-023-09345-3.

## Background

With population aging, the number of people with cognitive disorders is expected to increase. The most common cause of cognitive disorder is Alzheimer’s disease (AD) [1]. Cognitive disorders have significant social and economic impacts for the affected person, family, caregivers and society. In previous studies, higher use of healthcare resources of persons with AD has been shown to begin before the diagnosis, with a peak increase in costs one year before AD diagnosis [2, 3] Although associations between multimorbidity and dementia [4, 5], age of multimorbidity onset and dementia [6], and specific comorbidities and risk of AD (e.g. [7–10]) have been reported, it is not known which diagnosis categories contribute to the differences in healthcare service use and consequent cost increase before AD diagnosis, as to our knowledge there are no earlier studies that have assessed the distribution of diagnoses for healthcare service use before AD diagnosis.

AD has a preclinical phase before the symptoms fulfil the diagnostic criteria. It has been hypothesized that cognitive decline lags pathophysiological changes up to 15 years [11]. There is no cure for AD, but an intervention targeting modifiable risk factors has been shown to maintain cognitive functioning in persons at risk [12]. This pinpoints the need to recognize at-risk individuals in the preclinical phase. Although the increased utilization of healthcare resources prior to AD diagnosis [2, 3], higher prevalence of specific comorbidities among persons with AD (e.g. 7–10) and contribution of comorbidities to increased care costs after AD diagnosis [13, 14] have been demonstrated, to our knowledge it is still not known how broader diagnostic entities, such as different chapters of the International Classification of Diseases (ICD) contribute to accumulation of hospitalizations and outpatient visits prior to diagnosis.

Investigating the ‘why and when’ of specialized healthcare use of persons who are proceeding towards clinically verified AD can increase our understanding on the reasons for increased healthcare costs before AD diagnosis, but also on how at-risk individuals could be identified. Therefore, we investigated the relative contribution of hospitalizations and specialized healthcare outpatient visits from different diagnosis categories, based on the ICD-10 chapters, to overall specialized healthcare use during a ten-year period before AD diagnosis.

## Methods

This study is part of Medicine use and Alzheimer’s disease (MEDALZ) study, described in detail previously [15]. MEDALZ includes 70,719 Finnish persons who received incident, clinically confirmed AD diagnosis, indicated by special reimbursement to anti-dementia medication, and were community-dwelling at the time of diagnosis. The persons were diagnosed by either geriatrician or neurologist to have AD consistent with the National Institute of Neurological and Communicative Disorders and Stroke and the Alzheimer’s Disease and Related Disorders Association (NINCDS-ADRDA)[16] and The Diagnostic and Statistical Manual of Mental Disorders (DSM-IV)[17] criteria. The criteria require exclusion of alternative diagnoses and brain imaging with magnetic resonance imaging or computed tomography.

This study includes 42,934 persons diagnosed with incident AD between 1998 and 2011. For each of them, a matched comparison person without AD was identified on the AD diagnosis date (index date) from a register of Social Insurance Institution that contains information on persons eligible for reimbursed healthcare. The comparison persons (N = 42,934) were matched by sex and hospital district (both exact matches) and age (+/- one year) on the index date. Comparison persons were required not to have AD diagnosis, never purchased anti-dementia medications before the index date and within 12 months after the index date and to be alive and community-dwelling during the last day of the month of the index date.

Data on inpatient stays from primary and specialized healthcare and specialized healthcare outpatient visits were obtained from the Care Register for Healthcare using personal identification numbers. MEDALZ study protocol was approved by the register maintainers and all data were pseudonymized before submission to the research team. As the outpatient data are available since 1998, the assessment period was restricted to ten years before the index date. To illustrate temporal changes, this ten-year period was divided into twenty 6-month time windows.

The inpatient stays and outpatient visits (stays/visits) were grouped based on the main diagnosis of visits and main discharge diagnosis of stays, which were recorded using International Classification of Diseases 10th edition (ICD-10) of year 2011 [18]. We grouped the diagnoses at the chapter level with minor modifications (Supplementary Table 1). Briefly, diagnoses related to dementia from mental and behavioral, and from diseases of the nervous system (F00-F03 dementia and G30 Alzheimer’s disease) were grouped as “dementia”. Chapters with pregnancy, childbirth, puerperium and conditions originating in perinatal period were grouped together and due to their small amount (10 stays or visits in the AD cohort and 42 in the comparison cohort), they were excluded from the figures. Chapter including birth defects and chromosomal defects, and chapter including factors related to health status and contacts to healthcare were also combined. Outpatient visits with missing main diagnoses (109,488 visits for 23,597 persons with AD and 99,301 visits for 20,547 comparison persons) were included as their own category.

Statistical analyses were conducted using R, version 4.0.2. Descriptive statistics were calculated to describe the study population using means, proportions and 95% confidence intervals (CI). Odds ratio (OR) of having stays/visits per 6-month time windows between AD and comparison cohorts were computed with logistic regression with generalized estimation equations (GEE) using Huber/White/sandwich estimator of variance. We utilized GEE because we wanted to estimate population average. The model included AD and time (order of time window, range 1–20).

The proportion of persons with inpatient stay and/or outpatient visits was calculated in each 6-month time window. Both time window-specific and cumulative frequencies were derived. To investigate the relative distribution of diagnosis categories in each time window, the proportion of stays and/or visits in each category, relative to total number of stays/visits in the time window were calculated. These were visualized to observe changes in the relative contribution of diagnosis categories during the ten-year assessment period. To assess possible differences in the distribution of the diagnoses arising from year of AD diagnosis, sex or age at AD diagnosis (< 65, 65–74, 75–84, ≥ 85), we performed stratified sensitivity analyses. The absolute numbers of stays and visits in both cohorts were also visualized. Standardized mean differences, reported as Cohen’s d, were calculated to study differences in proportions of visits/stays in each diagnosis groups between AD and comparison cohorts.

## Results

The characteristics of study population are presented in Table 1. The mean age of the population on AD diagnosis date was 80.3 years and 64.7% were women. Nearly all people with AD (96.7%), and 91.1% of the comparison cohort had had hospital stay or outpatient visit during the 10-year assessment period.

**Table 1:** Characteristics of the Alzheimer’s disease and comparison cohorts on the index day (date of AD diagnosis)

Characteristic		Alzheimer’s disease cohort (n = 42 934)		Comparison cohort (n = 42 934)
**Age** mean (95% CI)		80.3 (80.3–80.4)		80.3 (80.3–80.4)
**Age groups** (n, %)				
<65		1 434 (3.3%)		1 439 (3.4%)
65–74		7 211 (16.8%)		7 206 (16.8%)
75–84		22 999 (53.6%)		22 999 (53.6%)
≥ 85		11 290 (26.3%)		11 290 (26.3%)
**Sex (n, %)**				
Men		15 139 (35.3%)		15 139 (35.3%)
Women		27 795 (64.7%)		27 795 (64.7%)
**Year of diagnosis**				
2008		9 335 (21.7%)		NA
2009		10 499 (24.5%)		NA
2010		10 878 (25.3%)		NA
2011		12 222 (28.5%)		NA
**Inpatient stay or specialized healthcare outpatient visit during the follow-up**				
Visit or stay		41 522 (96.7%)		39 095 (91.1%)
Visit		40 847 (95.1%)		38 307 (89.2%)
Stay		37 504 (87.4%)		34 709 (80.8%)

**Fig. 1:**
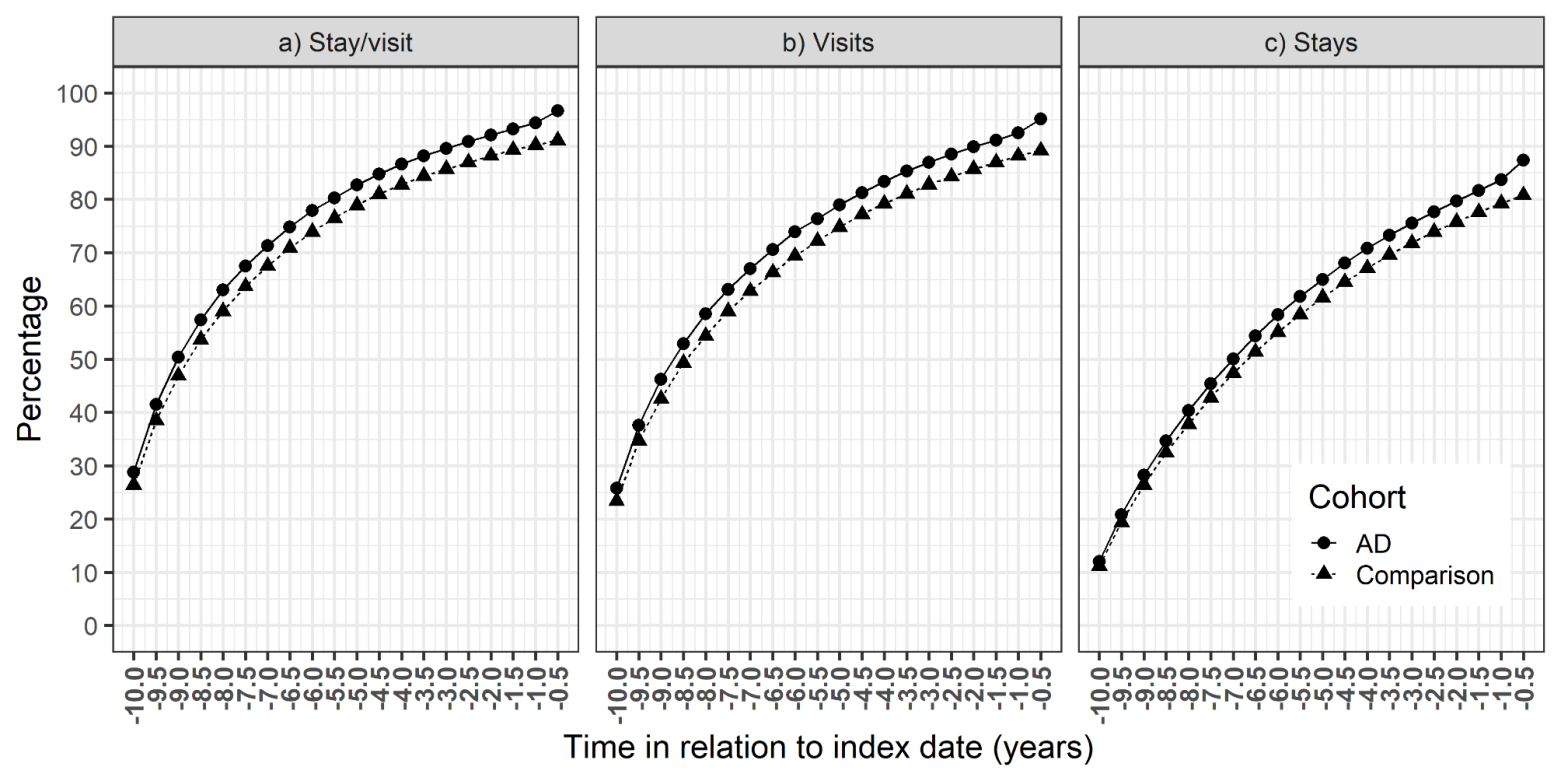
Cumulative proportions of persons with (a) either inpatient stay or specialized healthcare outpatient visit, (b) outpatient visit (c) inpatient stay in the Alzheimer’s disease (AD) cohort and comparison cohort during the 10-year assessment period before AD diagnosis

People with AD were more likely to have a stay/visit than the comparison cohort during the 10-year period (OR 1.19, 95% CI 1.17–1.21). Approximately half of both cohorts had stays/visits during the first 1.5 years of the 10-year assessment period, and the cumulative proportion increased until the end of the assessment period (Fig. 1a). The likelihood of a stay/visit increased over time (OR for time 1.04, 95% CI 1.042–1.044 per six-month increase), with a linear increase until the last three time windows and a steeper increase within the last 1.5 years before AD diagnosis, when the difference between AD and comparison cohorts was larger (OR, 95% CI 1.52, 1.49–1.55 for AD and 1.25, 1.24–1.26 for six-month-increase per time window during the last three time windows, respectively). Similar results were observed when inpatient stays (Fig. 1b) and outpatient visits (Fig. 1c) were investigated separately.

**Fig. 2:**
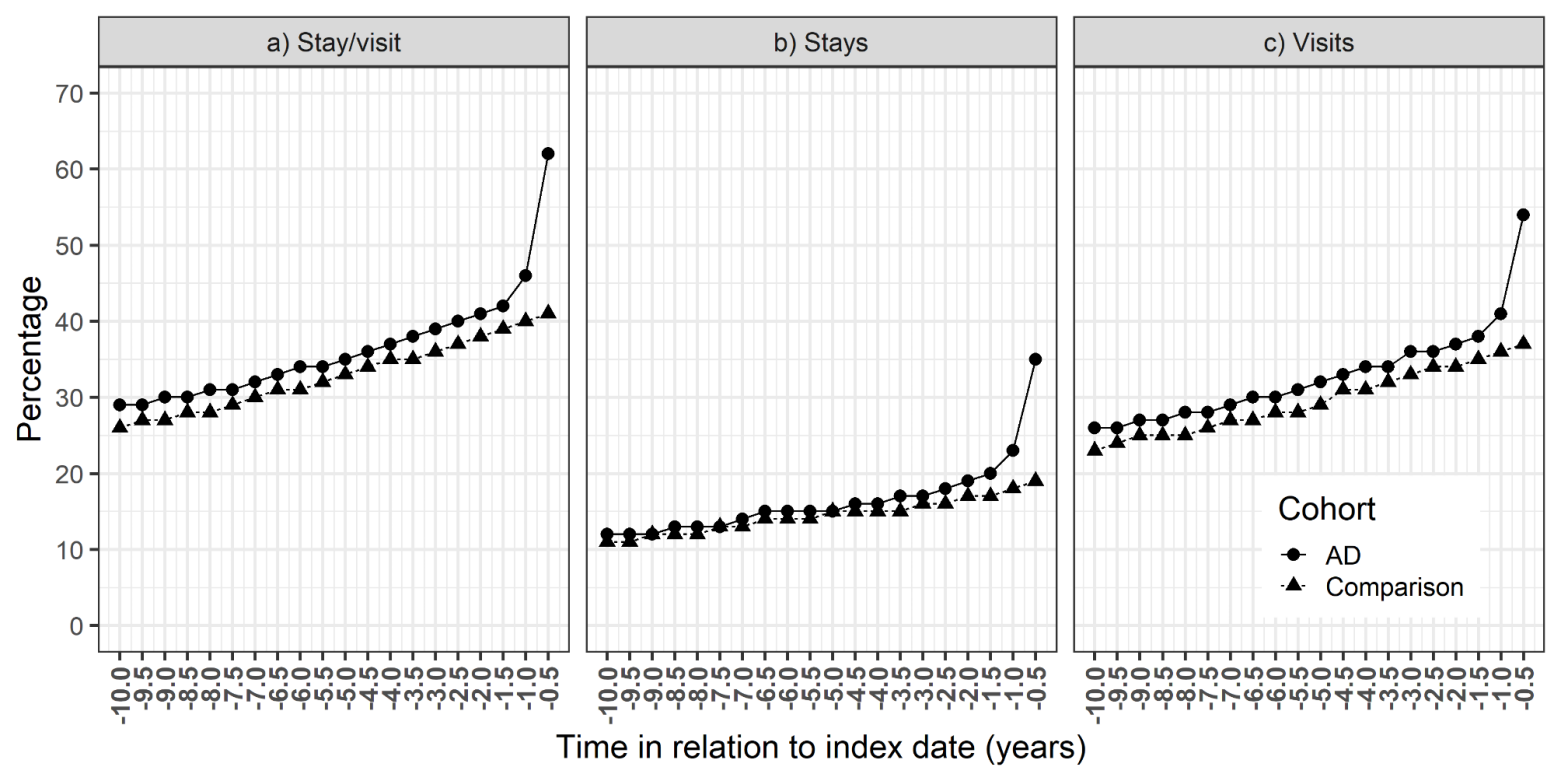
Proportions of persons with (a) either inpatient stay or specialized healthcare outpatient visit, (b) outpatient visit (c) inpatient stay in the Alzheimer’s disease (AD) cohort and comparison cohort in each 6-month time window during the 10-year assessment period before AD diagnosis

Less than 30% of the AD cohort had stays/visits during the first 6-month time window of the assessment period (i.e., -10 to -9.5 years before the index date), which increased to 62%, in the last time window (6 months before the index date, Fig. 2a). Prominent increase of stays/visits was observed one year before AD diagnosis in AD cohort but not in the comparison cohort. In the comparison cohort, the proportion of persons with stays/visits increased from 26 to 41% during the 10-year assessment period.

The proportion of persons with inpatient stays almost tripled in AD cohort (12–35%), while smaller increase occurred in the comparison cohort (11–19%, Fig. 2b). A constant increase in the number of persons with visits and/or stays was observed in both cohorts during the entire assessment period (Fig. 2a and c).

### Reasons for stays and visits

**Fig. 3:**
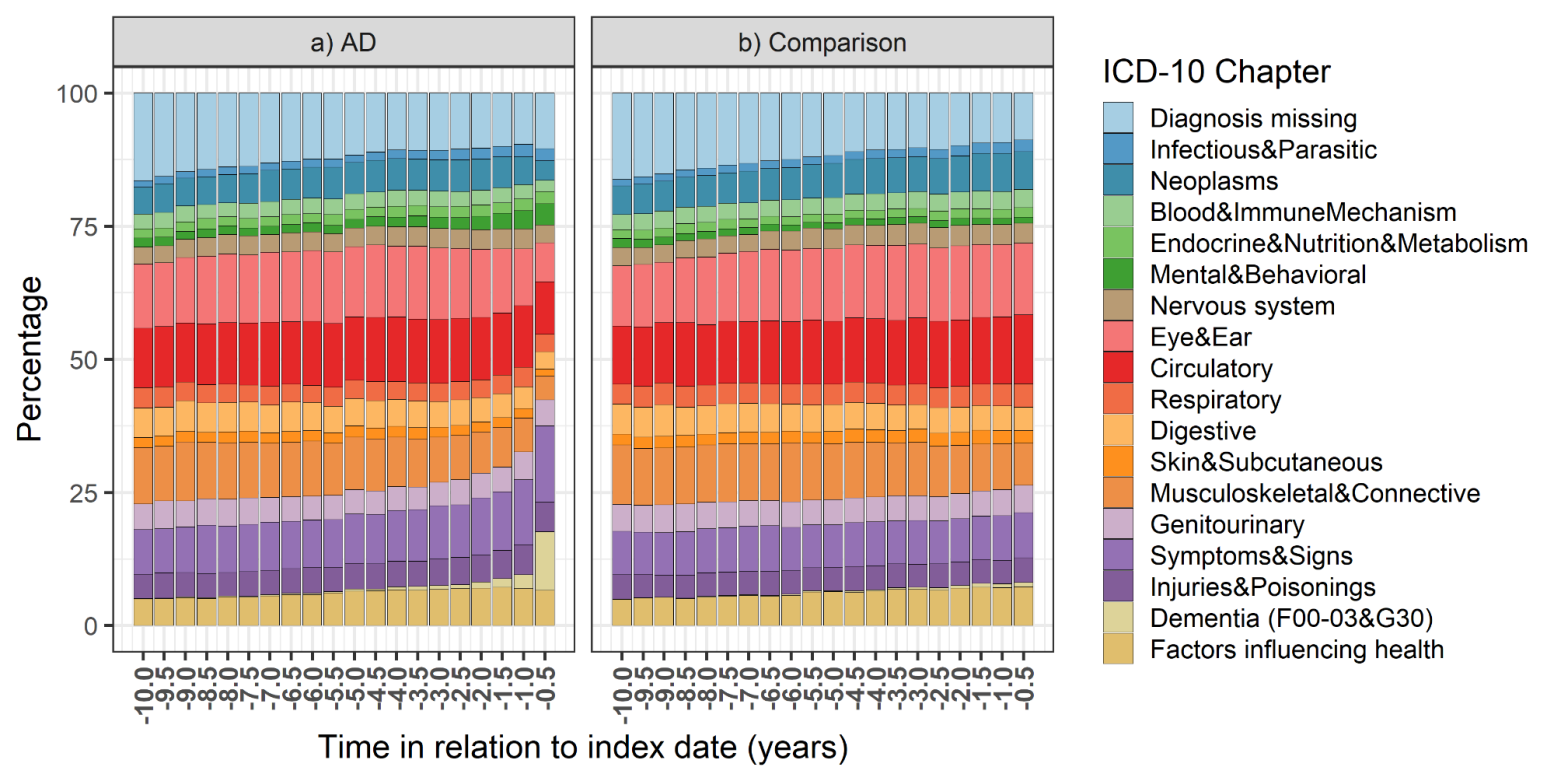
Relative proportions of different diagnosis categories from inpatient stays and specialized healthcare outpatient visits in (a) people with Alzheimer’s disease (AD) and (b) their comparison persons, in 6-month time windows during the 10-year assessment period

The relative proportions of different diagnosis categories in hospital stays and specialized healthcare visits were similar in both cohorts until approximately two years prior to index date (Fig. 3a and b). In both cohorts, the most common diagnosis categories were eye and ear disorders, diseases of the circulatory system and diseases of the musculoskeletal and connective tissue. In AD cohort, diagnoses of symptoms and signs, and mental and behavior disorders as the main diagnosis increased during the assessment period (Fig. 3a). The largest increase of stays/visits in these categories was observed in the last time window, i.e., 6 months before the index date, where the symptoms and signs were the most common diagnosis in the AD group.

The amount of outpatient visits from the symptoms and signs category, as well as those with missing diagnoses increased in AD cohort in the last time windows. The proportion of outpatient visits with missing diagnoses was highest in the earliest time window (16.5% and 16.2% in AD and comparison cohorts, respectively) and decreased during the assessment period, except for the last time windows in AD cohort.

In the AD cohort, dementia or AD as the main diagnosis for hospital stays and specialized healthcare visits became evident approximately four years before the AD diagnosis and the proportion of stays/visits from this diagnosis category increased noticeably in the last 6-month time window before the index date (Fig. 3a). There were also some persons in the comparison group who had hospital stays with dementia as the main diagnosis. However, the proportion of dementia stays from overall stays was very small in the control group. The largest differences between the AD and comparison cohorts were observed in this last time window, with largest standardized mean differences (Cohen’s d) observed in dementia (0.52), symptoms and signs (0.37), mental health (0.25) and missing diagnoses categories (0.23).

**Fig. 4:**
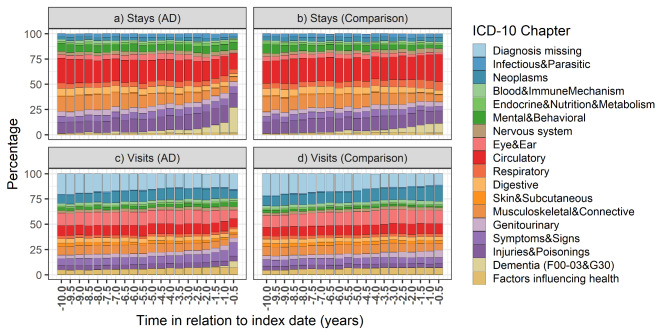
Relative proportions of different diagnosis categories from inpatient stays (a,b) and specialized healthcare outpatient visits (c,d) in persons with Alzheimer’s disease (AD, a,c) and b) their comparison persons (b, d), in 6-month time windows during the 10-year assessment period

When the relative contribution of different diagnosis categories to stays and visits were investigated separately, the results were similar to those obtained when visits and stays were combined (Fig. 4a, b, c and d). Furthermore, sensitivity analyses of the AD cohort were in line with the main analyses and did not demonstrate evident differences due to age at AD diagnosis (Supplementary Figure A1), year of AD diagnosis (Supplementary Figure A2) or sex (Supplementary Figure A3). When the absolute numbers of visits and stays were visualized, results were also similar (Supplementary Figure A4).

## Discussion

In our 10-year longitudinal nationwide study, the proportion of persons with hospital stays and specialized healthcare outpatient visits was very similar in persons with and without AD until 1.5 years before the diagnosis when an increase occurred in the AD cohort. Consistent with the earlier findings on increased healthcare costs of people with AD before the AD diagnosis [2, 3], the AD cohort had more visits and stays during the entire assessment period also in our study, although number of persons with stays/visits increased throughout the follow up in both cohorts. Our findings enrich earlier literature by systematically investigating how stays and visits in different diagnosis categories contribute to the overall healthcare use, and by illustrating how these change over time before the AD diagnosis. Our findings suggest that except for the last six months before AD diagnosis, the increased healthcare service use before AD diagnosis does not seem to arise from differences in specific diagnosis categories of ICD-10 such as diseases of the circulatory system, but from the higher frequency of visits and stays among persons with AD across diagnosis categories.

The estimated duration of preclinical AD is approximately 10 years, but it depends on several factors including age and apolipoprotein E genotypes, while the prodromal stage with amyloid accumulation and diagnoseable mild cognitive impairment is approximately four years [19]. Therefore, our assessment period period largely covers these pre-AD stages. It is likely that persons in the AD cohort did not have specific symptoms related to AD in the beginning of the assessment period. The early symptoms may pass unrecognized [20], and there might be a variety of unspecific symptoms including e.g. musculoskeletal disorders such as abnormal posture and unsteady gait, and neurological and psychological symptoms such as executive dysfunction and neuropsychiatric symptoms, which may occur before noticeable cognitive decline [20]. Therefore, as the stays/visits start to increase at the AD cohort already at the beginning of the assessment period, it is likely that these are due to these unspecific symptoms but also other comorbidities related to AD. The presence of unspecific symptoms is also supported by the increase in the symptoms and signs category, as this chapter of the ICD-10 includes “symptoms, signs, abnormal results of clinical or other investigative procedures, and ill-defined conditions regarding which no diagnosis classifiable elsewhere is recorded” [18].

AD is often comorbid with other somatic diseases such as type 2 diabetes and cardiovascular diseases, but also with, e.g., obesity and arthritis [10, 21]. These conditions have also been consistently associated with higher risk of AD. Therefore, although these are common conditions among older persons, the literature on risk factors implies higher prevalence in people with AD which would also explain the increased number of visits in the AD cohort. On the other hand, AD may trigger also psychiatric unrest including, e.g., depression and sleep disorders but also more severe psychiatric disorders including symptoms of schizophrenia and other psychosis. These symptoms may be present not only prodromal but also preclinical phase of AD and may contribute to increased number of stays/visits [10, 22].

In summary, we did not observe major differences in the relative contribution of diagnosis categories between persons with and without AD until the last 6-month time window before the index date. In that time window, differences between cohorts were observed in visits and stays with diagnosis categories for dementia, psychiatric diagnoses and symptoms of diseases. These, together with higher number of persons with missing diagnosis, and steepest increase in proportion of people with visits or stays, may reflect prodromal symptoms of AD and/or its diagnostic process. Regardless of the reason for original admission, symptoms of cognitive decline or confusion during the stay/visit might have aroused suspicion of cognitive disorder and led to referral for further examinations.

One strength of this study is the nationwide healthcare data, enabling the comparison of the proportion of people with and without AD with stays/visits. The participants’ AD diagnoses were clinically confirmed between 2008 and 2011, when the number of persons with incident special reimbursement was close to the estimated number of persons with incident AD diagnosis and initiation of anti-dementia medication is more common in Finland than in other countries [19]. During 2008–2011, anti-dementia medication was reimbursed in the mild and moderate phases of AD, so the persons with AD were likely in these phases on the AD diagnosis date, although data on AD severity were not available. It is possible, that some persons with AD were already in moderate phase on the date of diagnosis which may partially explain the differences observed in the last time windows. In addition, the contrast in our study was AD to no-AD, and therefore some of the persons in comparison cohort could have had dementia due to other causes. Although the main diagnosis was not recorded for all outpatient visits, nevertheless the proportion of visits with missing diagnosis was similar between the cohorts for most of the study period. Differences were observed only in the last time windows. Therefore, it is unlikely that the “missingness” would introduce bias, and the higher proportion on missing diagnoses in the last time window in the AD cohort may partially reflect the specificity of symptoms.

The generalizability of our findings may be affected by, e.g., cultural factors and differences in healthcare organization. The Finnish healthcare system is organized according to a national framework, and divided into primary and specialized healthcare. Primary healthcare services are provided at municipal health centers and refer to monitoring of the health of the population, promoting wellbeing and health and prevention, diagnosis and treatment of diseases, particularly public health diseases. Specialised healthcare refers to secondary and tertiary healthcare, provided by experts on medical specialities mainly in hospital settings [23]. All citizens and long-term residents are covered by tax-supported public health services, and they have access to health services regardless of socioeconomic status. Individual-level data on the use of healthcare services are collected to national registers, as this is mandated by law [24]. Cities and larger municipalities have public memory clinics. There was no age-based systematic cognitive screening in practice during the study period, although some municipalities arranged health checkups for residents at age 75 years. These checkups include cognitive screening with Mini-Mental State Examination. As these checkups were not organized by all communities, they did not cover the entire older population. The comparison cohort were matched by age and hospital district and therefore these checkups should have similar impact on both cohorts.

We emphasize that this study was intentionally performed on a cohort level, and although we did not observe differences on this general level, interindividual variation likely exists and should be studied further. We lacked information on the primary healthcare visits, and as many of the comorbidities of older adults are commonly treated in those settings, it would be interesting to perform a similar study with primary care visit data. Furthermore, we focused on diagnosis categories but did not have information on severity of diseases and their impact on participants’ health, functional ability or cognition. We used main diagnoses, which include those diagnoses clinicians considered to require the most effort in the stay/visit if there were several diagnoses.

### Conclusions and implications

Older adults have several medical conditions requiring treatment, and this is reflected in increasing number of visits and stays along with the increasing age, regardless the possible diagnosis of AD. However, persons with AD have more hospital stays and outpatient visits in specialized healthcare prior to their diagnosis of AD, which may reflect a variety of risk factors related to AD as well as a burden of multimorbidity in older population.

The difference in proportion of persons with and without AD, as well as the similarity of relative contribution of different diagnosis categories to specialized healthcare use between them implies that the increased healthcare service use before AD diagnosis is not so much arising from differences in specific diagnosis categories, but from higher contact frequency across diagnosis categories among persons with AD. Differences in diagnosis categories were observed only in the last time windows, when diagnosis categories of dementia, psychiatric diagnoses and symptoms and signs of diseases became more common in the AD cohort.

## Electronic supplementary material

Below is the link to the electronic supplementary material.

Supplementary Material 1

## Data Availability

The data that support the findings of this study are available from the corresponding author but restrictions apply to the availability of these data, and so they are not publicly available. Data are however available from the authors upon reasonable request and with permission of the register maintainers.

## Abbreviations

AD: Alzheimer’s disease
CI: confidence intervals
DSM-IV: The Diagnostic and Statistical Manual of Mental Disorders, 4th edition
GEE: generalized estimating equations
MEDALZ: Medicine use and Alzheimer’s disease study
NINCDS-ADRDA: the National Institute of Neurological and Communicative Disorders and Stroke and the Alzheimer’s Disease and Related Disorders Association
OR: odds ratio
