# Supplementary material for: Diagnostic groups of hospital stays and outpatient visits during 10 years before Alzheimer’s disease
Source: BMC Health Serv Res. 2023 Apr 4;23:339. doi: 10.1186/s12913-023-09345-3 (PMC10074798; doi:10.1186/s12913-023-09345-3)
Supplement: Supplementary file 1 — Supplementary Material 1 [file 12913_2023_9345_MOESM1_ESM.pdf]

Supplementary Table 1 International Classification of Diseases 10th edition chapters used in the study and analyses

Chapters of ICD-10 Classification	Name of the Chapter	Modified Categorizations Used in the Analyses	Shorter Name used in figures
1, A00-B99	Certain infectious and parasitic diseases		Infectious & parasitic
2, C00-D48	Neoplasms		Neoplasms
3, D50-D89	Diseases of the blood and blood-forming organs and certain disorders involving the immune mechanism		Blood & immune mechanism
4, E00-E90	Endocrine, nutritional and metabolic diseases		Endocrine & nutrition & metabolism
5, F04-F99*	Mental, Behavioral and Neurodevelopmental disorders	Excluding dementia in Alzheimer's disease (F00), vascular dementia (F01), dementia in other diseases elsewhere classified (F02) and unspecified dementia (F03)	Mental & behavioral
6, G00-99*	Diseases of the nervous system	Excluding Alzheimer's disease (G30)	Nervous system
5,6 F00-03 G30		Including F00-F03 and G30	Dementia
7&8, H00-H95	Diseases of the eye and adnexa, and diseases of the ear and mastoid process		Eye & ear
9, I00-I99	Diseases of the circulatory system		Circulatory
10, J00-J99	Diseases of the respiratory system		Respiratory
11, K00-K93	Diseases of the digestive system		Digestive
12, L00-L93	Diseases of the skin and subcutaneous tissue		Skin & subcutaneous
13, M00-M99	Diseases of the musculoskeletal system and connective tissue		Musculoskeletal/ connective
14, N00-N99	Diseases of the genitourinary system		Genitourinary
15&16, O00-P96	Pregnancy, childbirth and the puerperium, and certain conditions originating in the perinatal period		Pregnancy & puerperium
17, Q00-Q99<br>21, Z00-Z99	Congenital malformations, deformations and chromosomal abnormalities<br>Factors influencing health status and contact with health services		Factors influencing health
18, R0-R99	Symptoms, signs, abnormal clinical and laboratory findings not elsewhere classified		Symptoms & signs
19, S00-T98	Injury, poisoning and certain other consequences of external causes		Injuries & poisonings
		If main diagnosis of visit was not recorded	Diagnosis missing

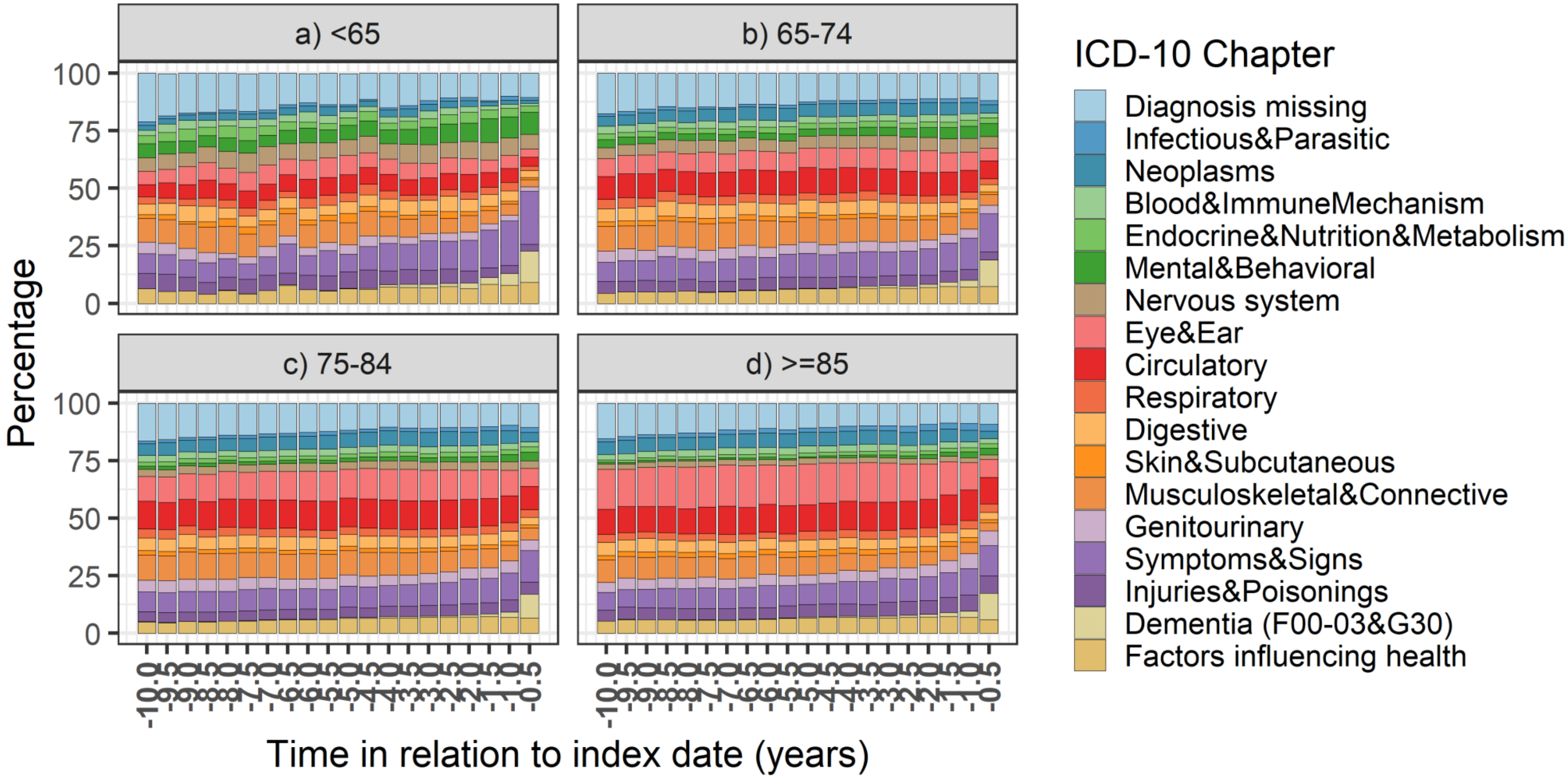

Supplementary Figure A1. Relative proportions of different diagnosis categories from inpatient stays and specialized healthcare outpatient visits in people with Alzheimer's disease in 6-month time windows during the 10-year follow-up before the AD diagnosis stratified by age at AD diagnosis.

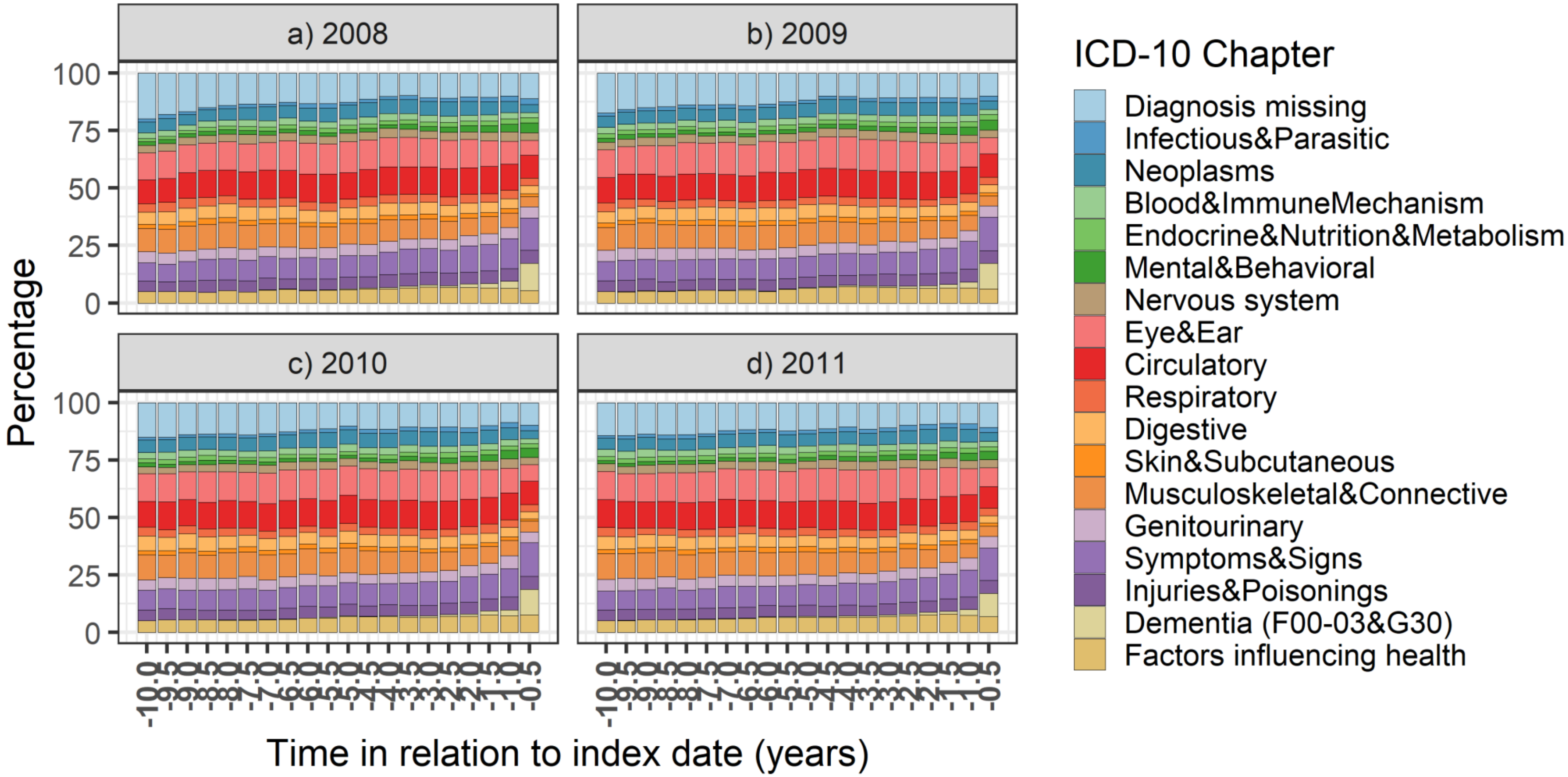

Supplementary Figure A2. Relative proportions of different diagnosis categories from inpatient stays and specialized healthcare outpatient visits in people with Alzheimer's disease in 6-month time windows during the 10-year follow-up before the AD diagnosis stratified by year of AD diagnosis

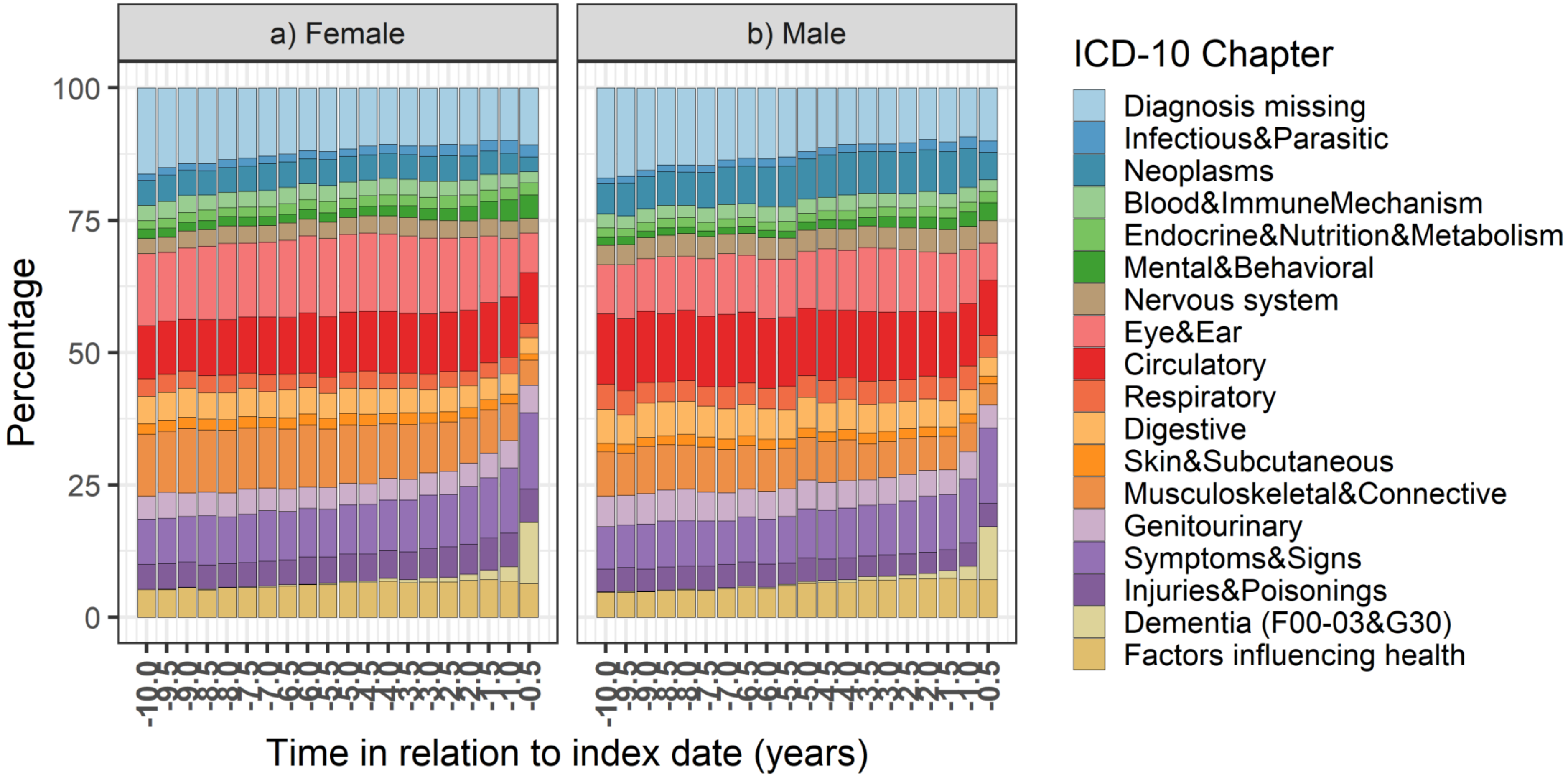

Supplementary Figure A3. Relative proportions of different diagnosis categories from inpatient stays and specialized healthcare outpatient visits in people with Alzheimer's disease in 6-month time windows during the 10-year follow-up before the AD diagnosis stratified by sex.

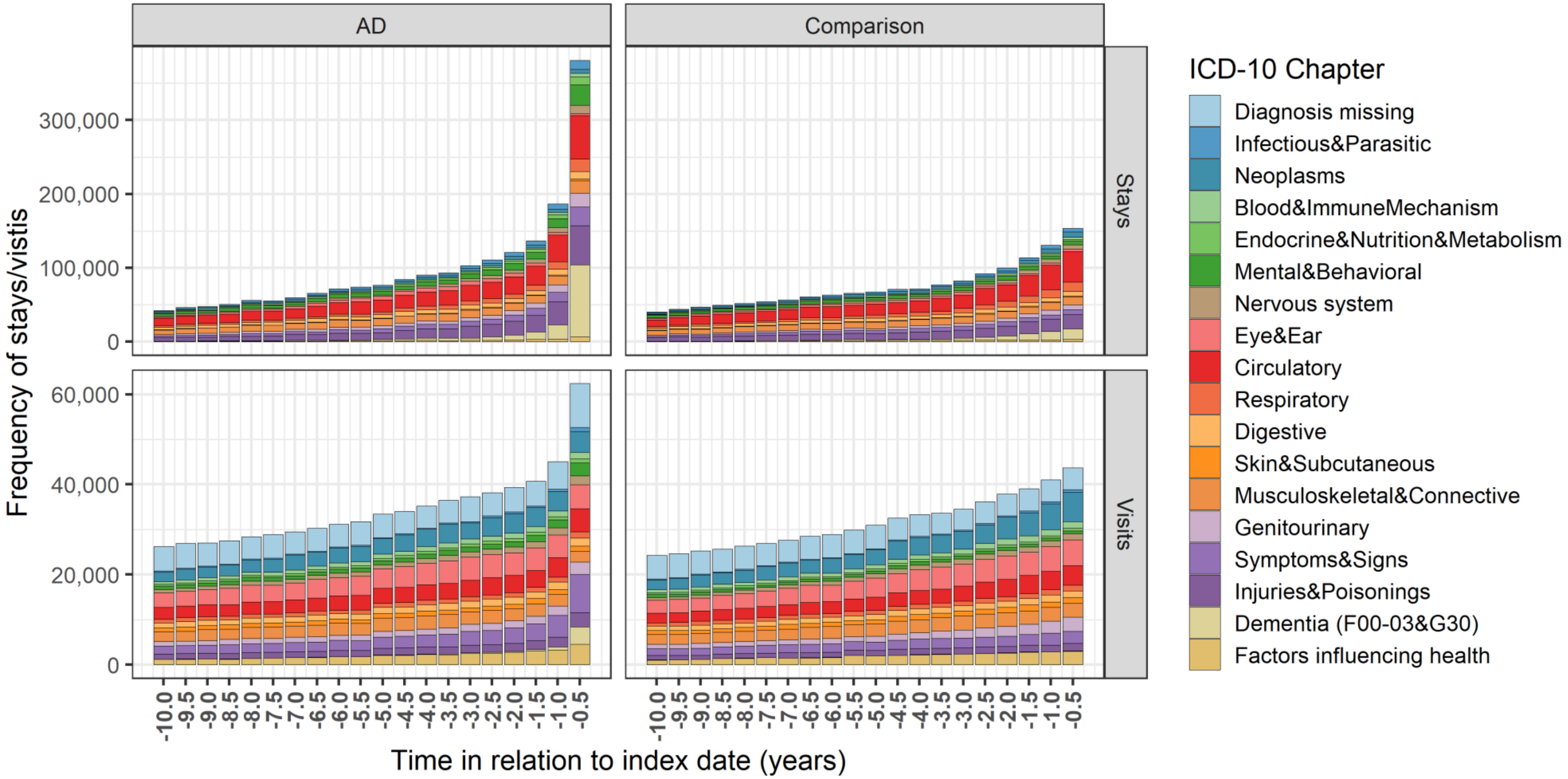

Supplementary Figure A4. Absolute number of inpatients stays (a,b) and specialized healthcare outpatient visits (c,d) from different diagnosis categories in persons with Alzheimer's disease (a,c) and their comparison persons (b,d) in 6-month time windows during the 10-year follow-up (cf. Figure 4)
